# Mpox virus: virology, molecular epidemiology, and global public health challenges

**DOI:** 10.3389/fmicb.2025.1624110

**Published:** 2025-07-17

**Authors:** Siwei Chen, Ju Huang, Junkai Chen, Fengqi Liu, Shuqi Wang, Na Wang, Ming Li, Ziyi Zhang, Congcong Huang, Weixing Du, Long Liu, Zhixin Liu

**Affiliations:** ^1^Department of Infectious Diseases, Renmin Hospital, School of Basic Medical Sciences, Hubei University of Medicine, Shiyan, China; ^2^Shiyan Key Laboratory of Virology, Hubei University of Medicine, Shiyan, China

**Keywords:** Mpox virus (MPXV), orthopoxvirus, molecular epidemiology, human-to-human transmission, public health challenges, vaccine equity, global health policy, surveillance gaps

## Abstract

Monkeypox (Mpox), a zoonotic disease caused by the Mpox virus (MPXV), has re-emerged as a significant global health concern, particularly since the 2022 outbreaks in non-endemic countries. MPXV shares close virological and genetic similarities with other orthopoxviruses, notably variola virus. The current circulating strains, primarily of clade IIb, exhibit enhanced human-to-human transmissibility. This review synthesizes recent advances in MPXV virology, pathogenesis, molecular evolution, clinical features, and diagnostic techniques. In addition, we highlight the mounting public health challenges, including vaccine inequity, immunity gaps in the post-smallpox era, surveillance limitations, healthcare system vulnerabilities, and stigma-related barriers to disease reporting and control. Addressing these multifaceted issues requires a globally coordinated response integrating equitable vaccine access, strengthened surveillance, stigma-free health education, and harmonized outbreak response strategies. A comprehensive understanding of these factors is critical to preventing future large-scale outbreaks and mitigating the global health burden posed by MPXV.

## 1 Virological characteristics of MPXV

**Figure 1:**
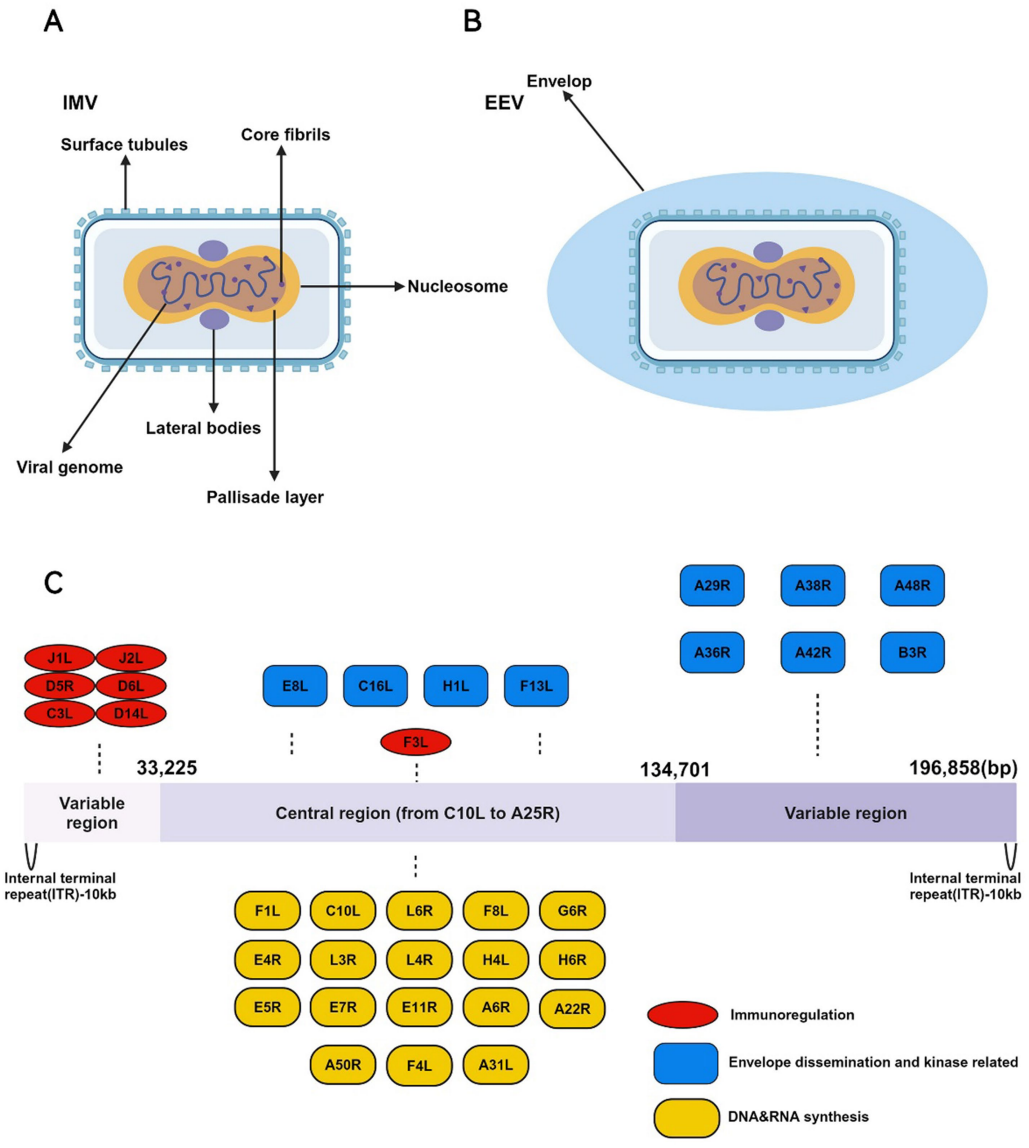
The IMV and EEV morphology and genomic structure of the MPXV. **(A)** The structure of IMV is highly complex and consists of the following key components: (1) Core envelope: The IMV is enclosed by a lipid bilayer embedded with surface tubules essential for entry and attachment to host cells. (2) Lateral bodies: Within the envelope, the IMV contains lateral bodies located on either side of the core. These lateral bodies store viral proteins that are released into the host cytoplasm early during infection, facilitating viral replication and immune evasion. (3) Viral core: The core is brick-shaped or ovoid, consisting of a proteinaceous outer layer surrounding the viral genome. Inside the core is the tightly packed, double-stranded DNA genome of approximately 197 kilobases (kb). The core also contains viral enzymes involved in DNA replication and transcription, including RNA polymerase, capping enzymes, and factors required for early gene expression. **(B)** The EEV form of MPXV is distinct from the IMV in that it has an additional outer lipid membrane. The EEV is primarily responsible for long-range dissemination within the host and between individuals. **(C)** The genome of MPXV is a double-stranded DNA genome, approximately 197 kilobases (kb) in length, making it one of the larger viral genomes. The genome structure is characterized by several key features: (1) Linear structure with inverted terminal repeats (ITRs): The genome contains inverted terminal repeats (ITRs), which consist of non-coding sequences and direct repeats. These regions play important roles in genome stability, replication, and resolution during the viral life cycle. (2) Central coding region: The central portion of the genome, comprising the majority of the coding sequence, is highly conserved among orthopoxviruses. It encodes essential genes involved in viral replication, transcription, and virion assembly, including those for DNA polymerase, RNA polymerase, capping enzymes, and structural proteins. This region is functionally crucial for the virus’s ability to replicate and produce infectious virions. (3) Variable regions: The regions flanking the central core are less conserved and contain genes that are non-essential for viral replication *in vitro*, but are critical for host-virus interactions, including immune evasion and virulence.

**Figure 2:**
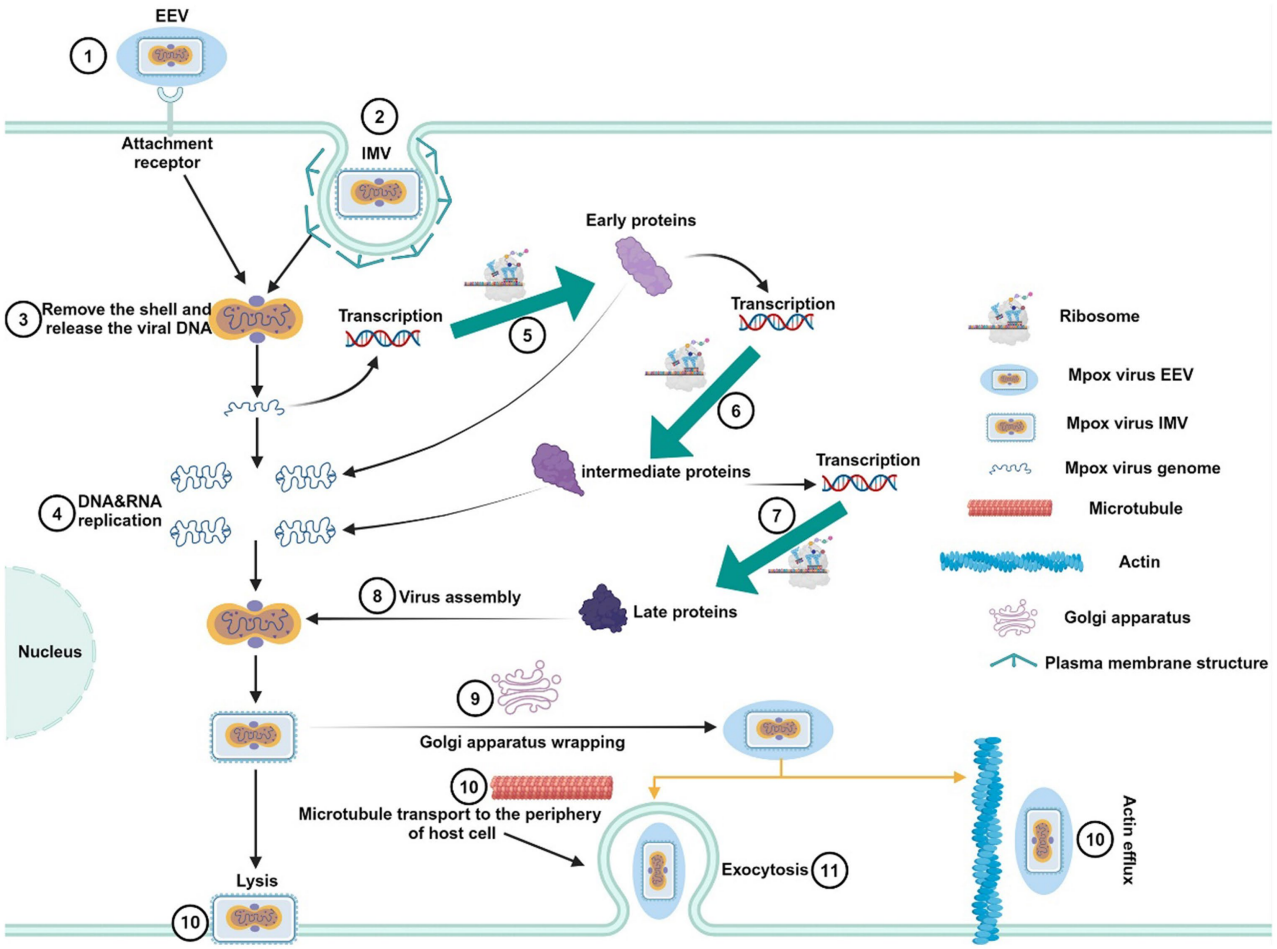
Replication life cycle of MPXV. First, EEV and IMV viral particles penetrate the host cell membrane via membrane fusion or endocytosis, releasing the viral core into the host cell cytoplasm. The released MPXV genome serves as a template for DNA replication and is transcribed to produce early proteins. These early proteins further facilitate DNA replication and the generation of intermediate transcription factors. Subsequently, intermediate transcription factors are transcribed and translated to form intermediate proteins, which promote the production of structural proteins, enzymes, and late transcription factors. The late transcription factors induce the synthesis of late proteins, which ultimately assemble into new viral particles. The newly formed IMV particles can be released through host cell lysis, or they can be enveloped via the Golgi apparatus to form EEV. EEV particles are then transported to the cell surface by actin or microtubules and secreted outside the cell.

MPXV belongs to the orthopoxvirus genus within the Poxviridae family. It is an enveloped linear double-stranded DNA virus, approximately 200–250 nm in size, appearing as oval or brick-shaped particles under electron microscopy, surrounded by a lipoprotein membrane (Jiang et al., 2023). The virus structure is composed of three main parts: the core, which contains the viral genome; the lateral bodies located on either side of the core; and the outer protein envelope. Although MPXV is a DNA virus, it primarily replicates in the cytoplasm of infected cells, encoding proteins that promote viral genome replication and gene expression (Alakunle et al., 2020). During its replication cycle, MPXV produces four distinct types of viral particles: intracellular mature virus (IMV), intracellular enveloped virus (IEV), cell-associated enveloped virus (CEV), and extracellular enveloped virus (EEV) (Yu et al., 2023). Among these, IMV and EEV are the primary infectious forms (Figures 1A,B) (Kmiec and Kirchhoff, 2022). The viral particles enter host cells through binding and fusion with the host cell membrane, after which they release pre-packaged viral proteins and enzymes into the cytoplasm, suppressing the host immune defense system and initiating early viral gene expression. The virus then undergoes DNA replication, transcription, and translation to complete its life cycle (Moss, 2013). Mature IMV can be released externally through cell lysis or by forming EEV via fusion with the Golgi apparatus and cytoplasmic membrane. EEV has two main release pathways: (1) transported along microtubules to the cell membrane, followed by exocytosis; (2) directly released into the external environment through interactions with actin (Figure 2) (Alkhalil et al., 2010). These distinct release mechanisms enable MPXV to efficiently spread within the host and accelerate its transmission in human populations.

MPXV, like variola virus (VARV), cowpox viruses (CPXV), and vaccinia virus (VACV), belongs to the orthopoxvirus genus and is one of the largest DNA viruses found in mammals. Phylogenetic analyses suggest that MPXV originated approximately 600 years ago in West Africa (Babkin et al., 2022). Genetic studies show that these four poxviruses share a common ancestor and have highly similar DNA sequences. Immunologically, these viruses exhibit cross-antigenicity, meaning infection with one of these viruses can provide some degree of protection against the other three (Diven, 2001).

In their study on orthopoxviruses, Shchelkunov et al. (2001) compared the amino acid sequences of MPXV and VARV, finding that the central genomic region of the two viruses has an amino acid sequence similarity of up to 96%, while the terminal regions show 83.5–93.6% amino acid similarity, indicating a close evolutionary relationship between MPXV and VARV. Additionally, research on the vaccinia virus tian tan strain (VTT) demonstrated that VACV and MPXV are structurally highly similar, with 90% homology in their DNA sequences, and they maintain this high degree of conservation during viral replication. Notably, VACV proteins A27L, L1R, D8L, B5R, and A33R show significant homology with MPXV proteins A29L, L1R, E8L, B6R, and A35R, respectively (Yang et al., 2023).

It is known that the VARV was one of the earliest poxviruses to cause disease in humans and is one of the most lethal orthopoxviruses. In the 17th century, people developed the practice of “variolation” which involved grinding the scabs from smallpox patients into a powder and blowing it into the nostrils of uninfected individuals to provide immunity against smallpox (Gopi et al., 2024). However, the mortality rate from this method was approximately 2%, presenting a significant risk. In the 18th century, to reduce the mortality associated with smallpox inoculation, Dr. Edward Jenner used pus from cowpox lesions to inoculate a boy. Although the boy developed mild symptoms, he fully recovered within a few weeks and did not contract smallpox when subsequently exposed to the virus (Willis, 1997). Jenner’s cowpox vaccination successfully provided protection against smallpox. Due to the cowpox vaccine being much safer than variolation, it quickly became the primary method for smallpox prevention (Jenner, 1894).

Over time, it was discovered that the VACV vaccine not only provided better protection against smallpox but was also safer. As a result, VACV gradually replaced CPXV as the primary vehicle for the smallpox vaccine. The VACV-based smallpox vaccine significantly reduced the risks associated with vaccination and played a crucial role in the global eradication of smallpox (Jacobs et al., 2009). In 1980, the World Health Organization (WHO) declared smallpox eradicated, after which China and other countries ceased smallpox vaccination. Consequently, individuals born after 1980 lack vaccine-induced immunity and are more susceptible to MPXV (Bunge et al., 2022). Studies indicate that the VACV-based smallpox vaccine offers approximately 85% protection against MPXV (Ahmed et al., 2022).

MPXV can be cultured in various cell lines, including rhesus monkey kidney cells and mouse liver cells. After inoculation with MPXV, its characteristic cytopathic effect (CPE) and plaque formation can be observed, with plaque size being a distinguishing feature between MPXV and VARV (McConnell et al., 1964). MPXV exhibits strong survival capabilities *in vitro*, showing good tolerance to drying and low temperatures, but it is sensitive to high temperatures. On contaminated surfaces, MPXV can survive for several months in cold environments (e.g., 4°C), but heating at 56°C for 30 min or 60°C for 10 min can inactivate the virus. MPXV is sensitive to common disinfectants and ultraviolet (UV) radiation, and it can be effectively inactivated by disinfectants such as sodium hypochlorite, glutaraldehyde, formaldehyde, and paraformaldehyde (General Office of National Health Commission, 2022).

## 2 Pathogenic mechanisms of MPXV

The MPXV genome is relatively large, approximately 197,000 base pairs (bp), encoding 190 open reading frames (ORFs) and comprising over 200 genes (Figure 1C) (Shchelkunov et al., 2002). The MPXV genome is divided into three main sections: a central region encoding essential enzymes and structural proteins required for viral DNA replication, transcription, and assembly; and two flanking regions containing inverted terminal repeats (ITRs), which are primarily involved in virus-host interactions, immune evasion, and apoptosis signaling (Gong et al., 2022).

Current research on MPXV focuses on immunogenic proteins, such as A33R, A37R, B4R, and B5R proteins (Table 1). The A33R protein facilitates the binding of the virus to host target cells (Suleman et al., 2022). The A37R protein, homologous to the A35R protein in CPXV, may play a role in modulating the expression of MHC class II molecules on the surface of host antigen-presenting cells. This function has been observed in CPXV’s A35R protein, and MPXV’s A37R protein is hypothesized to exhibit similar activity (Rehm et al., 2010). The B4R and B5R proteins of MPXV have multiple functions, including inhibiting host cell proliferation, regulating hematopoiesis, modulating immune responses, and participating in host cell adhesion, signaling, and mRNA transcription (Shchelkunov et al., 2002). Understanding the structure and function of these viral proteins is crucial for addressing genetic changes that arise during periods of high viral transmission and for studying the virus-host immune system interactions. This knowledge also provides essential insights for developing new MPXV vaccines that can offer immunity against different viral strains and that are suitable for large-scale production.

**Table 1:** Proteins encoded by MPXV and their functions.

Protein name	Gene name	Protein function	References
A33R	OPG161	Promote viral binding, fusion, and entry into target cells	Suleman et al. (2022)
A37R	OPG163	MHC class II antigen presentation inhibitor	Shchelkunov (2012)
A38R	OPG164	IEV transmembrane phosphoprotein	Hatmal et al. (2022)
A40L	OPG167	Integral membrane glycoprotein, Ig-like, regulates influx of extracellular Ca^2+^	Shchelkunov et al. (2002)
A41L	OPG170	Chemokine binding protein	Shchelkunov (2012)
A42R	OPG171	Profilin domain protein	Shchelkunov et al. (2002)
A43R	OPG172	Recognition of the antigen and the immune effects	Shchelkunov et al. (2002)
A45L	OPG174	Regulation of hormone synthesis and metabolism	Chen et al. (2005)
A46R	OPG175	Copper/zinc superoxide dismutase	Shchelkunov et al. (2002)
A47R	OPG176	Inhibition of the effects of IL-1	Chen et al. (2005)
A49R	OPG178	Thymidylate kinase	Shchelkunov et al. (2002)
A50R	OPG180	DNA ligase	Hatmal et al. (2022)
B4R	OPG189	Inhibit the host cell proliferation, regulate the immune response	Shchelkunov et al. (2002)
B5R	OPG190	Involved in host cell adhesion, signaling, as well as mRNA transcription	Shchelkunov et al. (2002)
B7R	OPG191	Recognition of the antigen and the immune effects	Chen et al. (2005)
B9R	OPG193	Involved in protein folding and assembly	Shchelkunov (2012)
B10R	OPG195	Intracellular viral protein, Inhibition of lymphocyte apoptosis	Shchelkunov (2012)
B11R	OPG198	Ser/Thr kinase, involved in intracellular signaling	Chen et al. (2005)
B13R	OPG200	Inhibition of the IL-1-binding proteins	Chen et al. (2005)
B16R	OPG204	IFN-alpha/beta-receptor-like secreted glycoprotein	Shchelkunov (2012)
B17R	OPG205	Involved in host cell adhesion, signaling, as well as mRNA transcription	Chen et al. (2005)
B19R	OPG208	It can antagonize serine protease activity	Shchelkunov (2012)
C1L	OPG039	Inhibit host cell apoptosis and participate in host cell adhesion	Chen et al. (2005)
C2L	OPG040	Antagonizing serine protease activity and inhibit host cell fusion	Chen et al. (2005)
C4L	OPG042	Participate in host cell lipid metabolism, biofilm formation	Shchelkunov et al. (2002)
C5L	OPG043	Involved in diverse host cell signaling and regulates cell proliferation	Chen et al. (2005)
C6R	OPG044	Inhibition of IL-1-binding protein function	Shchelkunov et al. (2002)
C7L	OPG045	Inhibition of host cell apoptosis	Shchelkunov (2012)
C8L	OPG046	Deoxyuridine triphosphatase production	Hatmal et al. (2022)
C9L	OPG047	Involved in protein ubiquitination modification	Shchelkunov et al. (2002)
C10L	OPG048	Ribonucleotide reductase small subunit	Shchelkunov et al. (2002)
C11L	OPG049	Recognition of the antigen and the immune effects	Chen et al. (2005)
D1L	OPG015	Involved in host cell adhesion, signaling, as well as mRNA transcription	Chen et al. (2005)
D3R	OPG115	Regulation of cell proliferation and differentiation	Chen et al. (2005)
D5R	OPG117	Zinc binding, virulence factor, inhibits UV-induced apoptosis	Shchelkunov et al. (2002)
D6L	OPG022	Secreted IL-18-binding protein	Shchelkunov et al. (2002)
D7L	OPG023	RNA polymerase subunit RPO18	Shchelkunov et al. (2002)
D9L	OPG025	Involved in host cell adhesion, signaling, as well as mRNA transcription	Shchelkunov et al. (2002)
D10L	OPG027	Regulating target cell enzyme activity and inhibit host cell apoptosis	Chen et al. (2005)
D12L	OPG124	Involved in protein ubiquitination modification	Shchelkunov et al. (2002)
D13L	OPG125	Inhibiting IL-1 action, preventing IL-1 from binding to the receptor	Chen et al. (2005)

The pathogenic mechanisms of MPXV are similar to other orthopoxviruses, such as VARV and VACV, in that MPXV is capable of infecting a variety of mammalian cells and is highly transmissible. MPXV’s initial infection targets primarily the oral cavity and respiratory tract, with an initial asymptomatic phase (Zaucha et al., 2001). The virus then migrates by infecting nearby immune cells and spreads from the primary infection site to adjacent draining lymph nodes. The infected immune cells may include monocytes, macrophages, B cells, and dendritic cells. There is debate regarding the early dissemination mechanisms of orthopoxviruses. For instance, in CPXV-infected mice, viral dissemination occurs through dendritic cells, transferring the virus from the pulmonary epithelium to the draining lymph nodes (Beauchamp et al., 2010). In contrast, VACV infection of human monocyte-derived dendritic cells impairs their maturation and migration potential, allowing the virus to evade immune responses, suggesting that VACV may not rely on dendritic cells for viral spread (Engelmayer et al., 1999).

In 1969, Wenner et al. (1969) studied the pathogenesis of MPXV by inoculating cynomolgus monkeys with the virus. They found that MPXV proliferated within cells at the injection site, accompanied by a local inflammatory response, including cell necrosis and vasculitis. MPXV was also detected in the lymph nodes and blood vessels, indicating that the virus spreads through lymphatic and blood pathways. MPXV reaches the lymphoid tissues in the neck and throat via draining lymph nodes, where it replicates, leading to viremia. Subsequently, the virus further disseminates through the bloodstream to the liver and spleen. Studies have shown that the liver and spleen are major targets for MPXV replication and dissemination. In these organs, viral infection exacerbates viremia, enabling further spread to the lungs, kidneys, and skin, which leads to various clinical symptoms in the host (Chapman et al., 2010).

In the early stages of MPXV infection, EEV viral particles interact with the primary attachment receptors on the host cell membrane—glycosaminoglycans (GAGs)—via their surface proteins to enter the host cell (Weaver and Isaacs, 2008). The specific host cell surface receptors and their associated proteins are not yet fully understood, but three MPXV-encoded proteins have been identified as being involved in viral entry. The first is the E8L protein, which can bind to chondroitin sulfate proteoglycans (CSPGs) on the host cell surface, mediating the adsorption of IMV to host cells (Weaver and Isaacs, 2008). The second is the L1 protein, a viral envelope protein. Studies on the pathogenesis of VACV have shown that the L1 protein is a key factor in the fusion of the viral envelope with the host cell membrane and is part of the entry/fusion complex (EFC). The L1 protein of MPXV shares high homology with that of VACV and likely plays a similar role in MPXV invasion of host cells, although this mechanism requires further investigation (Foo et al., 2009; Bisht et al., 2008). The third is the H3L envelope protein, which has been found to play an important role in viral adsorption to host cells and the formation of IMV in studies of VARV (Lin et al., 2000). In addition, MPXV evades the host immune system through multiple mechanisms during human infection. The virus can escape detection by host pattern recognition receptors (PRRs) that sense RNA, suppress IFN-mediated antiviral responses, control the apoptosis of infected cells, and limit the host’s inflammatory response. These mechanisms enhance viral replication capacity, creating a microenvironment conducive to viral replication, infection, and transmission (Yu et al., 2021; Zhu et al., 2023; Kindrachuk et al., 2012; Gupta, 2023).

## 3 MPXV epidemiology

Since the eradication of smallpox in the 1980s, orthopoxviruses had largely faded from public attention. However, with the global outbreak of MPXV, this virus and other orthopoxviruses have returned to the spotlight. Mpox, a zoonotic disease caused by MPXV, was first identified in 1958 in a laboratory in Copenhagen, Denmark, where a smallpox-like disease was observed in captive cynomolgus monkeys, giving rise to the name “monkeypox” (Ladnyj et al., 1972).

In 1970, MPXV was first isolated from a suspected smallpox patient in the Democratic Republic of the Congo (DRC). Between 1971 and 1978, 47 confirmed Mpox cases were recorded, the majority of which were in Central and West Africa, with 38 cases in the DRC. The remaining nine cases occurred in Cameroon, the Central African Republic, Gabon, Côte d’Ivoire, Liberia, Nigeria, and Sierra Leone (Breman et al., 1980). In May 2003, the first outbreak of MPXV outside of Africa occurred in the United States, with 72 suspected cases reported, of which 47 were confirmed in the laboratory. An investigation by the Centers for Disease Control and Prevention (CDC) linked this outbreak to prairie dogs imported from Africa (Kantele et al., 2016; Reed et al., 2004). From 2017 to 2018, Nigeria experienced a large-scale Mpox outbreak, with cases spreading to non-endemic countries (Sadeuh-Mba et al., 2019). In 2019, Singapore reported the first imported case of Mpox in Asia (Ng et al., 2019).

**Figure 3:**
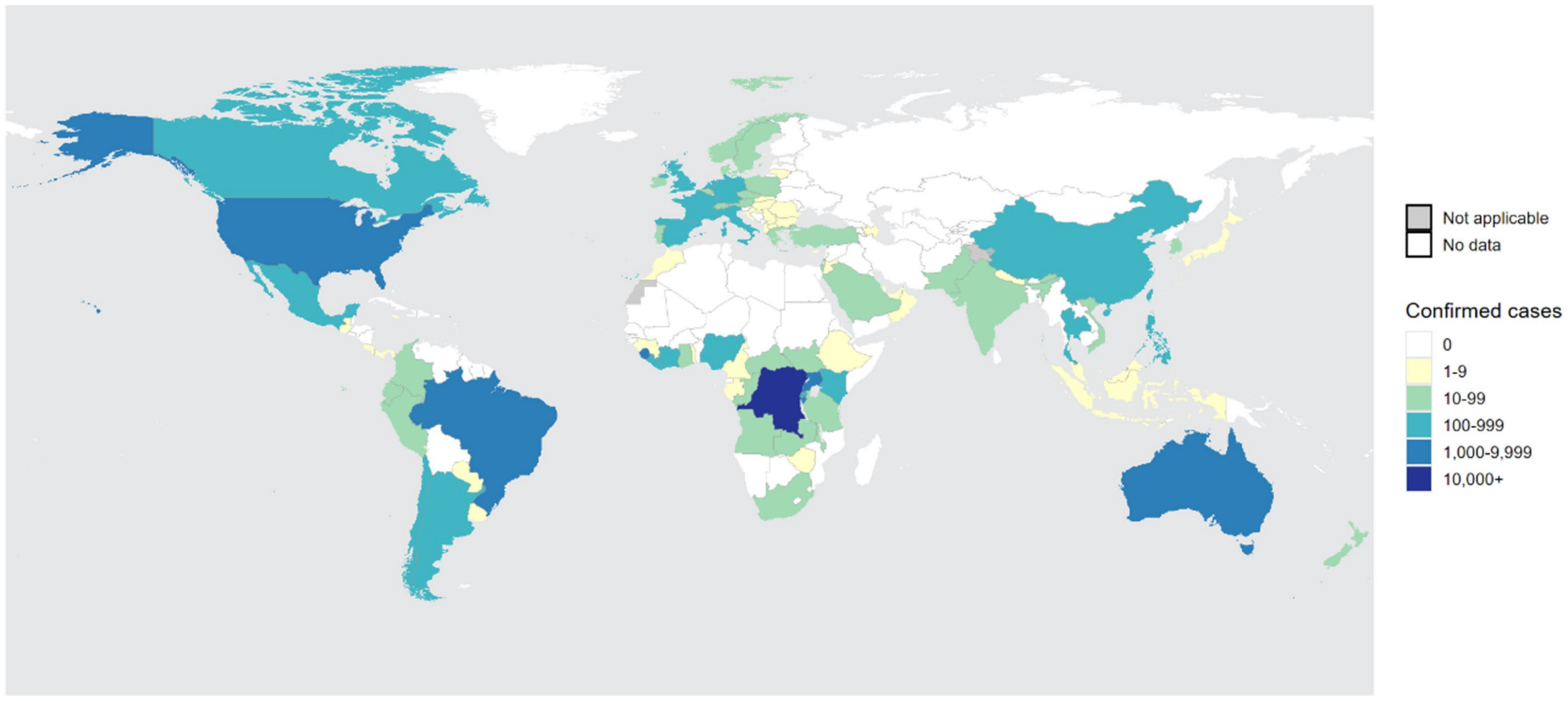
The global distribution of confirmed Mpox cases over the past year. From the figure, it is evident that Central Africa and West Africa remain high-incidence regions for Mpox. Furthermore, the number of infections in non-endemic regions, such as Americas, is experiencing a rapid upward trend, making it the second-largest area for Mpox infections after Africa. The statistical data is sourced from the World Health Organization (WHO) and covers the period from 31 May 2024 to 31 May 2025 (World Health Organization, 2025).

Before 2022, although a few non-endemic countries reported sporadic Mpox cases, these cases were relatively infrequent compared to those occurring in Africa during the same period. On May 16, 2022, the United Kingdom reported its first imported case of Mpox from Nigeria (Bunge et al., 2022). Subsequently, other non-endemic countries, including Spain, Portugal, Sweden, Canada, and Australia, also reported cases. By the end of May 2025, 133 countries and regions had reported a total of 150,069 Mpox cases to the World Health Organization (WHO), including 328 deaths, confirming that the Mpox outbreak is gradually becoming globalized (Figure 3). On July 23, 2022, the WHO declared the Mpox outbreak a “Public Health Emergency of International Concern (PHEIC)” and assessed the risk level as moderate. From January 2022 to May 2025, Europe and Americas reported a total of 99,432 confirmed Mpox cases, far exceeding the 42,829 cases reported in Africa. The top five regions reporting Mpox cases globally were the Americas (69,234 cases), Africa (42,829 cases), Europe (30,189 cases), the Western Pacific (5,869 cases), and Southeast Asia (1, 038 cases) (World Health Organization, 2025).

The first imported case of Mpox in China was reported in Taiwan in June 2022. In September of the same year, Hong Kong and Chongqing also reported their first imported cases, followed by multiple provinces reporting Mpox infections (Yang et al., 2022; Chiu et al., 2023; Zhao et al., 2022). This Mpox outbreak spread rapidly and widely, with many cases lacking travel histories to Central or West Africa, becoming a significant public health concern and attracting heightened attention from various countries and the WHO.

Genomic sequencing studies indicate that MPXV has two main evolutionary branches: Type I (Central African Congo Basin branch) and Type II (West African branch), both of which represent strains that initially infected humans. As MPXV continues to evolve, these branches have further diversified. The West African branch is divided into two sub-branches, IIa and IIb, with the IIb sub-branch further subdivided into multiple lineages, including A.1, A.1.1, A.2, B.1, and B.1.1. The strains responsible for the Nigerian outbreak in 2017–2018 were primarily of the A.2 lineage, while the imported strains reported in Singapore, the UK, the USA, and Israel from 2018 to 2021 were mainly of the A.1 lineage. The strains associated with the 2022 outbreak were predominantly of the B.1 lineage and its branches (Luna et al., 2022; Chakraborty et al., 2022).

Research indicates that strains from Branch I exhibit stronger virulence and transmissibility, with an average mortality rate of up to 10.6%. In contrast, strains from Branch II show lower virulence and transmissibility, with an average mortality rate of approximately 3.6% (Bunge et al., 2022). Geographically, Branch I strains are primarily prevalent in regions such as the Congo, Cameroon, and Sudan in Africa, whereas Branch II strains have spread across the continent, leading to intercontinental transmission and resulting in Mpox outbreaks in non-endemic countries like the United States and the United Kingdom.

Some researchers have conducted genomic comparisons between the B.1 lineage strains found in non-endemic countries like the United States and Germany in 2022 and the MPXV strains circulating in Nigeria from 2017 to 2019. They discovered approximately 50 single nucleotide polymorphism (SNP) sites of difference, with an SNP mutation rate more than six times that of other poxviruses, suggesting that the B.1 strain has a higher mutation rate and evolutionary speed (Isidro et al., 2022).

From the MPXV strains first identified in 1970 to the currently circulating strains, evolution has primarily manifested at the genetic level. MPXV continuously evolves through mechanisms such as point mutations, gene deletions, and recombination. For instance, the virus utilizes the host’s apolipoprotein B mRNA-editing enzyme catalytic polypeptide-like 3 (APOBEC3) cytidine deaminase to induce conversions of cytosines to uracils in foreign DNA during the viral replication process, leading to point mutations that facilitate the formation of new lineages (Gigante et al., 2022).

Epidemiological studies demonstrate that the virulence of MPXV varies across different evolutionary branches. The median lethal dose (LD50) of the West African branch is 1.29 × 10^5^ PFU per animal, while that of the Central African branch is 5.9 × 10^3^ PFU per animal, indicating higher virulence in the Central African branch. In animal experiments, prairie dogs inoculated with different doses of the Central African strain exhibited high morbidity and mortality rates, whereas those inoculated with the West African strain survived with minimal disease symptoms, providing experimental evidence for the differences in virulence between the two branches (Xiao et al., 2005; Hutson et al., 2010).

At the genomic level, the nucleotide sequence similarity between the Central African and West African branches is 99.5%, while the amino acid sequence similarity is 99.4%. The most significant genetic differences between the two branches are observed in the BR-203, BR-209, and COP-C3L genes. BR-203 is a homolog of the Myxoma virus (MYXV) M-T4 gene, which encodes a 221 amino acid protein. During MYXV infection, M-T4 primarily resides in the endoplasmic reticulum (ER), enhancing viral virulence and inhibiting apoptosis in infected cells (Hnatiuk et al., 1999). In MPXV Branch I, BR-203 encodes the full 221 amino acid protein; however, due to two base pair deletions in Branch II, it only encodes an N-terminal fragment of approximately 51 amino acids, leading to premature termination of translation and weakening the gene’s functionality (Bratke et al., 2013).

The BR-209 gene encodes a 326 amino acid protein that functions as an IL-1β-binding protein, preventing IL-1β from binding to its receptor and inhibiting the inflammatory response. In MPXV Branch I, the BR-209 gene encodes a complete protein consisting of an N-terminal 210 amino acids and a C-terminal 126 amino acids. In Branch II, a single base insertion near the N-terminal and a four-base deletion result in the encoding of only a fragment comprising an N-terminal 163 amino acids and a C-terminal 132 amino acids (Weaver and Isaacs, 2008; Chen et al., 2005). It remains unclear whether this difference in N-terminal length is related to the virulence differences between the Central and West African branches and requires further investigation.

The COP-C3L gene encodes a 263 amino acid complement control protein, referred to in MPXV as the Mpox complement inhibitor (MOPICE), which encodes a protein of 216 amino acids (Liszewski et al., 2006). This protein inhibits the complement pathway through a mechanism similar to mammalian complement activation regulators (RCA) (Blue et al., 2004). Although the complement control protein of MPXV has some base pair deletions, it still retains certain complement inhibitory functions (Liszewski et al., 2006). However, the COP-C3L gene is not expressed in Branch II, which may be one of the reasons for the differences in virulence between the two branches. Research has shown that integrating MOPICE into Branch II did not fully elevate its virulence to that of Branch I, but it altered the disease manifestation and accelerated disease progression (Hudson et al., 2012).

**Figure 4:**
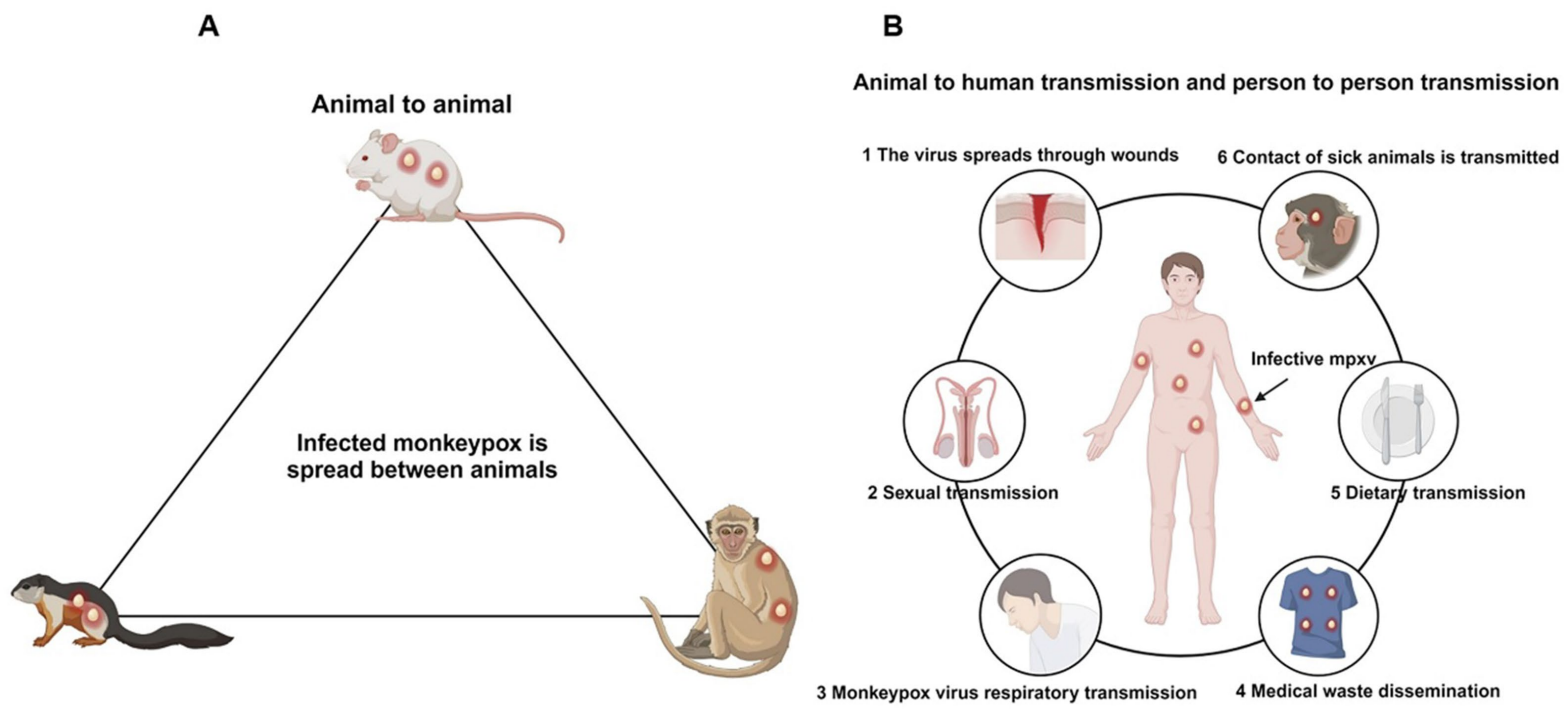
Transmission pathways of the Mpox. **(A)** The primary hosts of the Mpox are rodents, followed by primates, which serve as natural reservoirs. **(B)** Routes of human infection with the Mpox include wound contact, sexual transmission, respiratory transmission, contact with contaminated items, dietary transmission, and zoonotic transmission.

MPXV primarily spreads through rodent species such as Gambian pouched rats and dormice, with humans and monkeys acting as incidental hosts; therefore, this virus is classified as a zoonotic disease (Reynolds et al., 2012). The transmission of MPXV occurs through two main routes: animal-to-human and human-to-human transmission (Figure 4) (Seang et al., 2022). Animal-to-human transmission occurs through direct contact with the blood, bodily fluids, or lesions of infected animals. Human-to-human transmission primarily happens through contact with lesions, scabs, saliva, or other bodily fluids from infected individuals, as well as through contact with contaminated objects, such as clothing or towels that have not been disinfected (Guarner et al., 2022).

Although MPXV is not a typical sexually transmitted disease, viral DNA has been detected in semen, indicating its potential for sexual transmission (Antinori et al., 2022). In the context of modern open societal environments, the risk of sexual transmission of MPXV may heighten public awareness of sexually transmitted diseases, thereby reducing the occurrence of high-risk sexual behaviors.

Additionally, MPXV can be transmitted vertically to the fetus through the placenta during pregnancy and can also be transmitted to newborns during or after delivery through close contact (Kisalu and Mokili, 2017). A study conducted in the Democratic Republic of the Congo reported four pregnant women infected with MPXV, with only one newborn being born healthy (Mbala et al., 2017). While vaccination against smallpox is not recommended for pregnant women, infants in regions where Mpox is endemic should receive the smallpox vaccine promptly to prevent infection. Further research is needed to assess the potential impact of MPXV on pregnant women and appropriate response measures (Sookaromdee and Wiwanitkit, 2023).

Epidemiological reports indicate that past Mpox cases have primarily occurred in children and adolescents. From 1970 to 1989, the majority of Mpox infections in Africa were among children, with a median age of infection of 4 years (Heymann et al., 1998). In contrast, during the periods of 2000–2009 and 2010–2019, the median age of infection increased to 10 years and 21 years, respectively, indicating a gradual rise in the age of onset over time (Nolen et al., 2016; Rimoin et al., 2010). By 2022, during the Mpox outbreak in non-endemic countries, 79% of cases occurred in adult males aged 18 to 44, with a median age of 36 years (22–51). This shift may be related to the interruption of smallpox vaccination programs (Perez Duque et al., 2022).

The smallpox vaccine provides up to 85% cross-protection against MPXV; however, since smallpox was declared eradicated in the 1980s, vaccination ceased for individuals born after 1980, resulting in increased susceptibility to MPXV among these populations. Additionally, studies have shown that the infection rates of MPXV are similar between males and females. However, during the Mpox outbreaks in 2022, many cases were concentrated within the men who have sex with men (MSM) community. Reports from the Centers for Disease Control and Prevention (CDC) indicated that the majority of Mpox cases in the 2022 outbreak were among gay men, significantly increasing the infection risk for specific populations, including homosexuals, bisexuals, and transgender individuals (Li et al., 2023; Khaity et al., 2022).

The immune function of Mpox -infected individuals is correlated with the severity of MPXV infection. Research has shown that HIV-positive individuals exhibit more pronounced symptoms after MPXV infection, including rashes, fever, genital ulcers, inguinal lymphadenopathy, and necrotic lesions in the perianal area, genitals, oral cavity, trunk, and face (Perez Duque et al., 2022; de Sousa et al., 2022). During the MPXV outbreak in Nigeria from 2017 to 2018, more than half of Mpox -related deaths occurred in patients co-infected with HIV (Yinka-Ogunleye et al., 2019). Co-infection with HIV and MPXV leads to prolonged disease courses, more severe lesions, difficulties in healing, and a higher incidence of secondary bacterial infections and genital ulcers (Ogoina et al., 2020).

A recent report documented cases of HIV-positive individuals co-infected with MPXV and syphilis, exhibiting extremely severe symptoms, including systemic Mpox manifestations along with necrosis of the nose and severe infections of the penis and oral mucosa (Boesecke et al., 2022). Therefore, individuals who are unvaccinated, engage in high-risk sexual behaviors, have compromised immune systems, are elderly, or are children should be prioritized for protection against MPXV infection (Petersen et al., 2019).

Currently, the predominant MPXV strains circulating globally (outside certain regions of Africa) are derived from the B.1 lineage, which is a micro-evolutionary branch of the IIb clade. This lineage is characterized by an enhanced adaptability to humans during its evolution, exhibiting stronger transmissibility but lower pathogenicity, with a mortality rate of less than 1% (Yu et al., 2023; Isidro et al., 2022). Epidemiological investigations have indicated that, since the cessation of smallpox vaccination, global immunity to orthopoxviruses has gradually declined, as evidenced by the increasing median age of Mpox cases. Early MPXV infections were primarily confined to Central and West Africa, with sporadic cases occurring in other continents, though their impact was minimal.

With the acceleration of globalization, the Mpox outbreak in 2022 demonstrated that MPXV has acquired new genetic mutations during its prolonged evolutionary process, leading to a significant increase in cases among individuals with no travel history to endemic regions. This indicates that MPXV has the potential to continue evolving and could trigger large-scale outbreaks similar to smallpox. Therefore, countries urgently need to implement existing preventive measures to actively curb the spread of Mpox.

## 4 Clinical manifestations of Mpox

**Figure 5:**
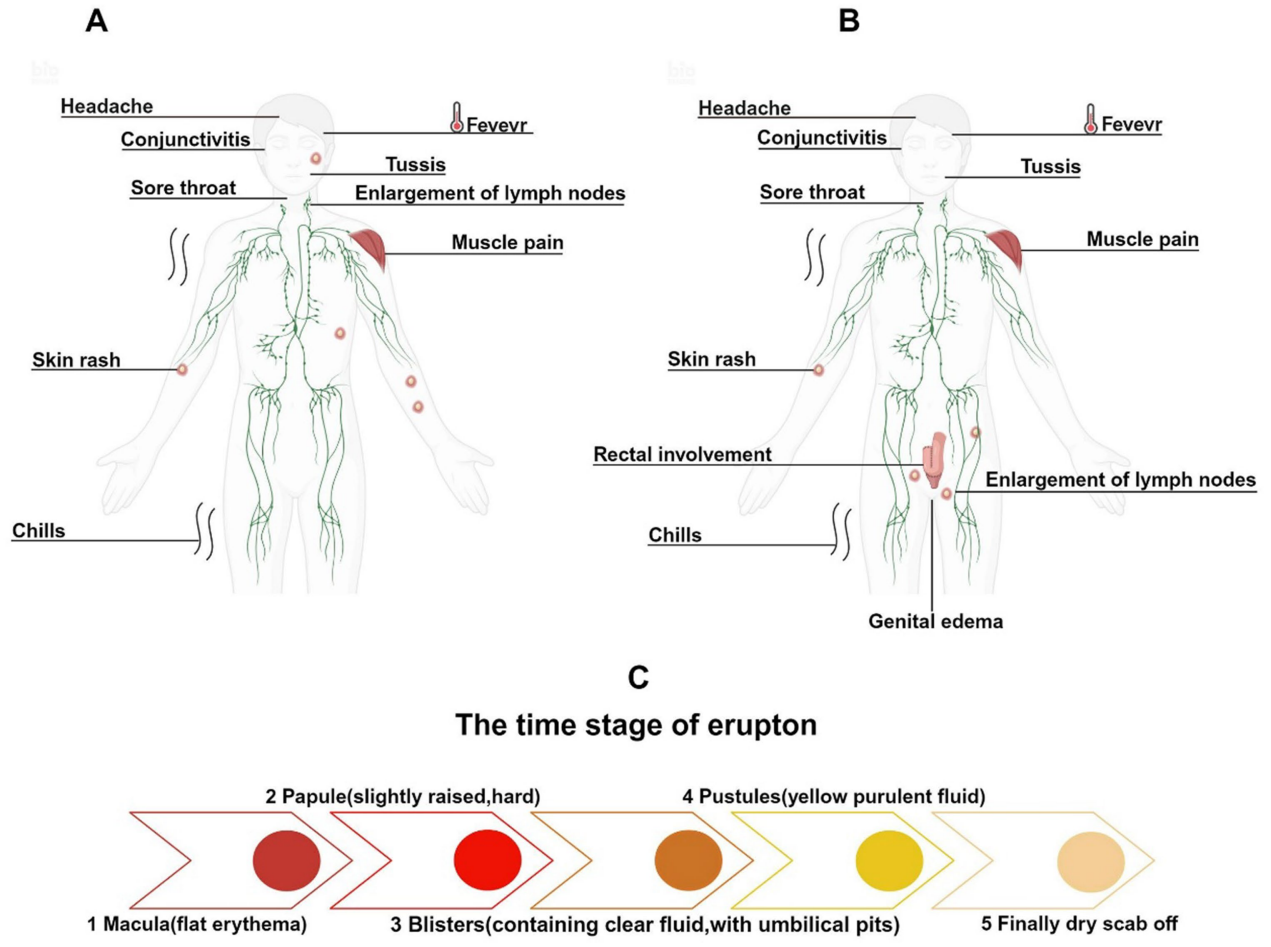
Clinical symptoms and rash evolution of Mpox infections across different historical periods. **(A)** The main clinical symptoms of early Mpox infections included facial rashes and cervical lymphadenopathy. **(B)** In the 2022 Mpox outbreak, rashes primarily appeared around the genital area, with lymphadenopathy first occurring in the groin. **(C)** The evolution of the rash.

MPXV infection presents similarly to VARV and is classified as a self-limiting disease. After infection, MPXV enters the host through various transmission routes and has an incubation period of 5 to 21 days. Upon completion of the incubation period, patients typically exhibit signs akin to those of viral infections, including fever, headache, lymphadenopathy, chills, fatigue, lethargy, and myalgia. After 1 to 3 days of fever, a rash usually begins to appear, starting on the face and then spreading to the trunk and limbs, forming large rashes that can number from a few to thousands. The evolution of the rash includes macules (flat red spots), papules (slightly raised, firm lesions), vesicles (fluid-filled lesions with a central dimple), pustules (containing yellow purulent fluid), and finally crusting and desquamation (Figure 5C) (Damon, 2011). The course of the disease typically lasts 2 to 4 weeks, after which the lesions generally heal, leaving atrophic scars (Sukhdeo et al., 2022).

In certain severe cases, complications may arise, including conjunctivitis, corneal infections, encephalitis, tonsillitis, pharyngitis, bronchopneumonia, syphilis, gonorrhea, herpes simplex, and genital Chlamydia infections (Okoli et al., 2023). Prior to 2022, among the circulating MPXV strains, male patients accounted for approximately 55%, slightly more than female patients. The rash was predominantly located on the head and neck (98%), trunk (94%), limbs (91%), and genital region (53.5%), usually exhibiting a centrifugal distribution and can be widespread (Figure 5A) (Li et al., 2023).

Although it has been reported that approximately 99% of cases during the 2022 Mpox outbreak occurred in male patients (Li et al., 2023), another study has indicated a substantial proportion of cases among women as well, accounting for 17.22% of infections (Satapathy et al., 2024). Overall, the 2022 Mpox outbreak disproportionately affected men compared to women. The incubation period was shorter, and rashes were primarily distributed in the genital area (55.6%), perianal area (39.8%), trunk (45.2%), limbs (41.8%), and head and neck (36.7%), with the perianal and genital regions often being the initial sites of rash development (Figure 5B) (Li et al., 2023). Furthermore, in the 2022 Mpox cases, about half of the patients’ rashes did not follow the typical progression, potentially skipping one or more rash stages. Most patients had fewer than 10 rashes, with 10% of patients presenting with only one rash. The rash at the initial site frequently appeared as a “doughnut-like” pseudopustule, characterized by raised edges and a central dimple (Catala et al., 2022; Patel et al., 2022).

Patients with compromised immune function, such as those infected with HIV or those with other immunosuppressive conditions, tended to have larger lesions with deeper inflammation, which could result in wart-like plaques or large ulcers, prolonged healing times, and significant scarring (Gonzalez-Torres et al., 2023; Saldana et al., 2023). In this outbreak, traditional prodromal symptoms such as fever, fatigue, and headache were milder and even absent, with these symptoms appearing not only before the rash but also during or after its onset (Hatami et al., 2023).

Lymphadenopathy also presented differently; while systemic lymphadenopathy was common in previous outbreaks, in this outbreak, it was often limited to one or two sites associated with the rash, and the swollen lymph nodes shifted from the cervical region to the groin (Okoli et al., 2023; Hatami et al., 2023). Additionally, patients who engaged in anal intercourse may exhibit rectal involvement, presenting with symptoms such as rectal-perianal pain, hematochezia, diarrhea, and tenesmus. In severe cases, ulcerative lesions may develop near the intestinal mucosa, leading to intestinal perforation (Patel et al., 2022; Mailhe et al., 2023; Tarin-Vicente et al., 2022). Due to inflammation potentially involving all layers of the skin, rashes often accompanied by edema could lead to significant swelling of the penis and scrotum when lesions appear in the male genital area. In severe cases, multiple rashes may coalesce, potentially affecting the urethra, leading to urinary difficulties and hematuria, which may require surgical intervention (Gomez-Garberi et al., 2022).

## 5 Diagnostic techniques for Mpox

Due to the extremely similar clinical presentations caused by different orthopoxviruses, it is challenging to diagnose Mpox solely based on infection symptoms. Therefore, diagnostic techniques with high sensitivity and rapid detection rates are crucial for controlling outbreaks. Currently, polymerase chain reaction (PCR) and real-time quantitative polymerase chain reaction (real-time PCR) are the conventional methods of choice for detecting suspected MPXV infections.

Samples of vesicular exudate and scab material are typically used to confirm MPXV infection. In PCR, MPXV is specifically identified by detecting an 8-bp deletion in the gene encoding the A-type inclusions (ATI) protein (Neubauer et al., 1998). Real-time PCR selects the conserved regions of the B6R, E9L, C3L, F3L, and N3R genes as amplification targets (Kulesh et al., 2004; Li et al., 2006; Maksyutov et al., 2016).

Other techniques, such as isothermal amplification, are efficient and rapid but may produce non-specific amplification, necessitating precise primer design; these methods are still under investigation (Feng et al., 2022). Virus isolation and culture are classical diagnostic methods, with MPXV growing well in cell lines such as HeLa and Vero, as well as in chick embryos (Erez et al., 2019). However, isolating and culturing MPXV must be performed in a biosafety level 3 (BSL-3) laboratory or higher, and should be conducted by experienced personnel. Despite the implementation of comprehensive personal protective measures, there remains a risk of infection (Hong et al., 2023).

Electron microscopy can identify viral particles in rashes, vesicles, and scabs. However, these particles appear similar to those of other orthopoxviruses under the microscope, indicating only that the virus belongs to the orthopoxvirus genus and not specifying the exact species. Moreover, electron microscopy requires a high level of knowledge from the operator, and there are certain infection risks associated with the sample preparation process (Gelderblom and Madeley, 2018). Enzyme-linked immunosorbent assay (ELISA) can detect specific IgM and IgG antibodies in the serum of Mpox patients 1 to 4 weeks after the appearance of rashes. However, due to its lower specificity, it is susceptible to interference from cross-reactivity with other orthopoxviruses (Zhou and Chen, 2023; Kabuga and El Zowalaty, 2019).

## 6 Treatment and prognosis of Mpox

During the 2003 Mpox outbreak in the United States, the CDC suggested that administration of the smallpox vaccine (SPX) within 2 weeks of infection might alleviate disease severity (Guarner et al., 2022). However, the vaccine was neither made publicly available nor routinely used in Mpox patients. This was largely due to high production costs relative to the limited number of cases, as well as concerns over safety. Live smallpox vaccines have been associated with serious adverse events in vulnerable populations, including cryptococcal meningitis, myocarditis, pneumonia, vision loss, and complications during pregnancy (Petersen et al., 2015).

For severe Mpox cases, more intensive interventions may be required. These include cross-protective vaccines such as ACAM2000, JYNNEOS (also known as IMVAMUNE or Imvanex), and Vaccinia Immune Globulin Intravenous (VIGIV), along with antiviral agents developed for smallpox—namely tecovirimat, cidofovir, and brincidofovir (Petersen et al., 2015; Grosenbach et al., 2023; Merchlinsky et al., 2019; Chan-Tack et al., 2021).

Currently, effective antiviral therapies specifically approved for the clinical treatment of Mpox remain limited. As a result, antiviral agents originally developed and stockpiled for smallpox—such as tecovirimat, cidofovir, and brincidofovir—are currently being repurposed for Mpox management under the protocols of the U.S. Strategic National Stockpile (SNS) (Centers for Disease Control and Prevention, 2025).

Tecovirimat (TPOXX), a small-molecule inhibitor targeting the VP37 protein on the surface of orthopoxviruses (Ferdous et al., 2022), is the first-line treatment for patients with severe or high-risk disease—such as immunocompromised individuals, pregnant women, and children (Gessain et al., 2022). It is available under an expanded-access investigational new drug (EA-IND) protocol. Although randomized trials (e.g., STOMP and PALM007) have demonstrated tecovirimat’s safety, they have not shown a significant reduction in lesion healing time. In cases of progressive disease, treatment failure, or suspected resistance, combination therapy with brincidofovir (an oral prodrug of cidofovir) or intravenous cidofovir is recommended, both accessible through emergency-use frameworks.

Cidofovir (CDV, [(S)-1-(3-hydroxy-2-phosphonomethoxypropyl) cytosine]), a nucleotide analog inhibiting viral DNA polymerase (Lea and Bryson, 1996), has demonstrated efficacy *in vitro* and in animal models but is limited in clinical use due to its nephrotoxicity. Brincidofovir (CMX001; BCV), a lipid-conjugated derivative of cidofovir approved in 2021 for smallpox (Harris et al., 2022), offers improved oral bioavailability and intracellular activation. It has shown survival benefit in preclinical models (Islam et al., 2023), although clinical data in Mpox remain sparse. Its adverse effects—including gastrointestinal disturbances and transient elevations in liver enzymes—necessitate careful monitoring during treatment. While it is better tolerated than cidofovir, brincidofovir has a less favorable safety profile than tecovirimat.

Vaccinia immune globulin (VIGIV) may be considered for immunocompromised individuals or those with ocular involvement, although its effectiveness in Mpox is not yet well established. Treatment decisions should be tailored to individual clinical scenarios and guided by CDC recommendations, especially in light of emerging antiviral resistance and the complexity of severe cases.

Mpox is generally a self-limiting disease, with most immunocompetent patients recovering within two to four weeks. Supportive care remains the cornerstone of management and includes antipyretics, analgesics, fluid and electrolyte balance, skin hygiene to prevent secondary infections, and antibiotics when indicated. However, in vulnerable populations—such as young children, pregnant women, immunocompromised individuals, and persons living with HIV—Mpox can progress to severe or even fatal outcomes. Prognosis in these groups hinges on prompt antiviral therapy and comprehensive supportive measures.

Despite progress in therapeutic options, several key challenges remain. First, high-quality randomized controlled trials (RCTs) are lacking, and current treatment recommendations are based primarily on observational data or preclinical studies. Second, viral evolution—particularly mutations in genes encoding VP37 or DNA polymerase—may reduce drug sensitivity. Third, access to antiviral agents remains uneven, especially in endemic and resource-limited regions, further exacerbating global health disparities. To overcome these limitations, ongoing research is exploring innovative approaches, including host-directed therapies, immunomodulatory drugs, and MPXV-specific monoclonal antibodies. Well-designed multicenter clinical trials and longitudinal cohort studies are urgently needed to determine treatment efficacy and establish standardized, evidence-based protocols for diverse patient populations.

## 7 Public health challenges of the Mpox outbreak

Since the global resurgence of Mpox in 2022, the disease has posed significant challenges to public health systems worldwide (World Health Organization, 2025). These challenges are multifaceted, encompassing issues of vaccine equity, disease surveillance, public communication, healthcare infrastructure, and international policy coordination. Understanding these challenges is critical to preventing future large-scale outbreaks and mitigating the public health burden of Mpox.

The cessation of smallpox vaccination after the disease’s eradication in 1980 has left a vast proportion of the global population without immunity to orthopoxviruses, including MPXV (Bunge et al., 2022; Breman et al., 1980). This immunity gap is particularly evident in individuals born after 1980, who now constitute the majority of the global adult population. Although vaccines such as JYNNEOS and ACAM2000 are available, access remains heavily skewed toward high-income countries (Petersen et al., 2015; Grosenbach et al., 2023; Merchlinsky et al., 2019; Chan-Tack et al., 2021). In contrast, many low- and middle-income countries, including those in Africa where Mpox is endemic, face considerable barriers to vaccine acquisition, including supply chain limitations, financial constraints, and regulatory delays. This inequitable distribution undermines global containment efforts and exacerbates the vulnerability of high-risk populations.

The 2022 outbreak revealed a disproportionate impact on men who have sex with men (MSM) (Li et al., 2023). While this has important epidemiological implications, it has also led to stigma and misinformation that hindered case reporting, contact tracing, and access to healthcare. In some regions, individuals avoided testing or treatment due to fear of discrimination. Public health messaging that is inclusive, nonjudgmental, and culturally sensitive is essential to address this issue. Furthermore, overemphasis on MSM populations risks obscuring other transmission routes and susceptible groups, potentially leading to incomplete surveillance.

Accurate and timely surveillance is a cornerstone of epidemic response. However, Mpox surveillance is still fragmented globally. Many endemic regions lack robust infrastructure for genomic sequencing, contact tracing, and rapid diagnostics. Even in non-endemic countries, the initial response to the 2022 outbreak was delayed due to a lack of familiarity with the disease and insufficient diagnostic capacity. Strengthening surveillance systems and integrating Mpox testing into broader infectious disease platforms are crucial for early detection and containment.

Many health systems, particularly in resource-constrained settings, are ill-equipped to manage additional outbreaks such as Mpox. During the COVID-19 pandemic, healthcare personnel and facilities were already strained, and the emergence of Mpox added further pressure. This is especially concerning in contexts with limited isolation wards, inadequate training on poxvirus infection control, and insufficient stockpiles of personal protective equipment (PPE) and antivirals. Integrating Mpox preparedness into broader epidemic response frameworks is essential to building resilience.

Although the World Health Organization (WHO) declared Mpox a Public Health Emergency of International Concern (PHEIC) in 2022, international responses were inconsistent (World Health Organization, 2025). Some countries instituted aggressive contact tracing and ring vaccination programs, while others delayed action or downplayed the threat. This lack of coordinated strategy reflects a broader challenge in global health governance—ensuring timely, equitable, and science-based responses across geopolitical boundaries. Future frameworks must emphasize international collaboration, resource sharing, and equitable policy implementation.

## 8 Conclusion

Although MPXV was first identified decades ago, its recent global resurgence—particularly since 2022—marks a new phase in its epidemiological significance. Genetic evolution, increased human-to-human transmissibility, and the absence of population-wide smallpox vaccination have contributed to widespread susceptibility. Our review highlights critical findings in MPXV virology, pathogenic mechanisms, and diagnostic tools, which are essential for guiding clinical and scientific responses.

However, beyond biomedical understanding, the current outbreak underscores profound public health challenges. The lack of equitable access to vaccines, persistent surveillance and diagnostic gaps, health system limitations in resource-poor settings, and social stigma—especially affecting MSM populations—have collectively hindered effective outbreak control. Moreover, fragmented international responses point to the urgent need for coordinated global strategies.

Going forward, successful containment of Mpox will depend not only on scientific innovation but also on inclusive public health policies, international cooperation, and investment in pandemic preparedness infrastructure. A unified, equity-centered approach is essential to ensure that MPXV does not evolve into a persistent and preventable global health threat.
